# Editorial: Interactions Between Proteins and Biomacromolecules: Tools and Applications

**DOI:** 10.3389/fmolb.2021.708084

**Published:** 2021-06-11

**Authors:** Qunye Zhang, Lianli Chi, Fuming Zhang

**Affiliations:** ^1^ The Key Laboratory of Cardiovascular Remodeling and Function Research, Chinese Ministry of Education, Chinese National Health Commission and Chinese Academy of Medical Sciences, The State and Shandong Province Joint Key Laboratory of Translational Cardiovascular Medicine, Department of Cardiology, Qilu Hospital, Shandong University, Jinan, China; ^2^ National Glycoengineering Research Center, Shandong University, Qingdao, China; ^3^ Rensselaer Polytechnic Institute, Troy, NY, United States

**Keywords:** interaction, biomacromolecule, protein, glycosaminoglycan, SARS-CoV-2

**Editorial on the Research Topic**

**Interactions Between Proteins and Biomacromolecules: Tools and Applications**

## Introduction

As the protagonist in the symphony of life, proteins perform fundamental functions by interacting with other biomacromolecules including nucleic acids, and carbohydrates. Therefore, the interactions between proteins and biomacromolecules have always been the pivotal issue in biomedical research. In the past 2 decades, advances in technologies including mass spectrometry (MS), next-generation DNA sequencing, and bioinformatics give birth to a new field: interactomics (Luck et al., 2017).

In this Special Issue, we selected a series of articles that highlight technological advances for studying interactomics and the interactomics of protein-glycosaminoglycans (GAGs) and protein-miRNA. We hope that this Special Issue will instigate novel questions in the minds of our readers and will be helpful in facilitating the development of the field.

## Technological Advances for Studying Interactomics

Characterization of interactions between proteins and biomacromolecules includes determining their selectivity or measuring their binding affinity. Selectivity is primarily screened by affinity chromatography, while binding affinity can be analyzed by a variety of technologies, such as isothermal titration calorimetry, surface plasmon resonance (SPR) and bio-layer interferometry. The advances of structural characterization technologies, such as MS and nuclear magnetic resonance (NMR) spectroscopy, make it possible to directly analyze the complexes formed by proteins and biomacromolecules. In this Special Issue, the technological advances for studying interactomics has been reviewed by Shi et al. A more specific review, focused on NMR characterization of GAGs and proteins, was provided by Bu and Jin. With the rapid accumulation of omics data, how to fully utilize these high-throughput data to uncover interactions between proteins and biomacromolecules has become a highly attractive research area (Hawe et al., 2019). Ding et al. presented a tool based on delayed comparison and Apriori algorithm to find protein-protein interactions according to the temporal information in time-series proteomic data. This tool is instrumental in fully utilizing the time-series omics data.

## Interactomics of Protein-GAG and Protein-miRNA

GAGs are a family of highly negatively charged linear polysaccharides including heparin/heparan sulfate (HS), chondroitin sulfate (CS)/dermatan sulfate (DS), and keratan sulfate (KS). GAGs play vital roles in many pathological and physiological processes, such as embryonic development, extracellular matrix assembly, inflammation, cancer, and cardiovascular diseases, by interacting with numerous proteins. The study of protein-GAG interactions is an important theme in glycobiology, resulting in many therapeutic implications. Recently, the first comprehensive draft of GAG interactome was reported, which composes of 932 protein-GAG interactions (Vallet et al., 2021).

In this Special Issue, Shi et al. present an excellent review on the progress of protein-GAG interactions research. Bu and Jin review the application of NMR spectroscopy on the characterization of protein-GAG interactions. Since the COVID-19 pandemic, it was reported that the interactions between cellular HS and S-protein of SARS-CoV-2 are critical for the viral infection (Kim et al., 2020 and Clausen et al., 2020). Yue et al. review the latest advances in HS–protein interactions studies related to COVID-19. In a research article, Yue et al. confirmed HS facilitates S-protein-mediated SARS-CoV-2 host cell invasion. In Alzheimer’s disease (AD) research, protein Tau (tau) and related tau pathology have been a hot research area since the interactions between tau and HS proteoglycans (HSPGs) are key facilitator in each stage of the prion-like propagation of pathology. Mah et al. review the sulfation code of HSPGs in tauopathies. Additionally, Kim et al. report the kinetics and structural features of heparin and its interactions with cellular prion protein measured by SPR.

MiRNAs are a class of RNA molecules with important regulatory functions (Ebert et al., 2012), but there are few studies on protein-miRNA interactions, especially about miRNA transport, distribution in organelles and secretion. In this Special Issue, Guo et al. discovered that MSI2 could bind to miR-301a-3p and facilitate its distribution in mitochondria using affinity purification and non-labeling proteomic techniques. This study provided valuable insight into the mechanism of mitochondrial distribution of miRNAs.

## Future Perspectives

This collection of articles highlights the different approaches for studying interactomics and related new analytical techniques. Indeed, the breakthrough in analytical tools (ultra-highly sensitive and automatic level) and approaches (such as machination learning) during the last decade has facilitated a greater understanding of the importance of interactions between biomacromolecules and their roles in diseases. We expect the more outcomes from this interdisciplinary research will accelerate the identification of new biomarkers for diagnostics and drug discovery.
